# Validation of the scandinavian guidelines for initial management of minor and moderate head trauma in children

**DOI:** 10.1007/s00068-019-01288-x

**Published:** 2020-01-06

**Authors:** Caroline Sönnerqvist, Ole Brus, Magnus Olivecrona

**Affiliations:** 1 School of Medical Sciences, Örebro University, Örebro, Sweden; 2 Clinical Epidemiology and Biostatics, School of Medical Sciences, Örebro University, Örebro, Sweden; 3 Department of Medical Sciences, Faculty of Medicine and Health, Örebro University, Örebro, Sweden; 4 Department of Anaesthesiology and Intensive Care, Section for Neurosurgery, Örebro University Hospital, Örebro, Sweden

**Keywords:** Traumatic brain injury, Children, CT, Guidelines, Validation

## Abstract

**Background:**

Head trauma in children is common, with a low rate of clinically important traumatic brain injury. CT scan is the reference standard for diagnosis of traumatic brain injury, of which the increasing use is alarming because of the risk of induction of lethal malignancies. Recently, the Scandinavian Neurotrauma Committee derived new guidelines for the initial management of minor and moderate head trauma. Our aim was to validate these guidelines.

**Methods:**

We applied the guidelines to a population consisting of children with mild and moderate head trauma, enrolled in the study: “Identification of children at very low risk of clinically-important brain injuries after head trauma: a prospective cohort study” by Kuppermann et al. (Lancet 374(9696):1160–1170, https://doi.org/10.1016/S0140-6736(09)61558-0, 2009). We calculated the negative predictive values of the guidelines to assess their ability to distinguish children without clinically-important traumatic brain injuries and traumatic brain injuries on CT scans, for whom CT could be omitted.

**Results:**

We analysed a population of 43,025 children. For clinically-important brain injuries among children with minimal head injuries, the negative predictive value was 99.8% and the rate was 0.15%. For traumatic findings on CT, the negative predictive value was 96.9%. Traumatic finding on CT was detected in 3.1% of children with minimal head injuries who underwent a CT examination, which accounts for 0.45% of all children in this group.

**Conclusion:**

Children with minimal head injuries can be safely discharged with oral and written instructions. Use of the SNC-G will potentially reduce the use of CT.

## Background

A fairly recent review estimates that in high-income countries, annually, 691/100,000 children attend emergency departments and 74/100,000 children are admitted to hospital because of a traumatic brain injury (TBI) [1]. A mild TBI (70–90% of all TBI) is usually defined by the Head Injury Severity Scale (HISS) as a patient conscious at first assessment, with GCS 14–15, that may have had a brief loss of consciousness (LOC) or amnesia but without any neurological deficits on admission [2, 3]. A minor part of all TBI can be considered as clinically important TBI (CITBI). These should be distinguished rapidly and precise, and for this, CT scan is the reference standard [4]. Children with minor head injuries account for 40–60% of all traumatic brain injuries examined with CT. Less than 10% of these show radiological signs of TBI [5, 6]. Of all children with mild TBI, the rate of CITBI is about 1%, the need of neurosurgery 0.2% and the rate of mortality even lower [2, 4].

CT scanning is the main contributor to ionising radiation to the population, and the annual number of CT examinations between the year 2000 and 2016 has been increased by 320% [7].

The rate of lethal malignancies from paediatric CT scans has been estimated to be between 1/1000 and 1/5000, with a higher risk in younger ages [8, 9]. With this in mind, and advocated by the Nordic Radiation Protection Authorities, CT scan should only be done when clinically justified.

Decision rules for head trauma management have recently been developed with the increasing CT use in consideration and in order to reduce the number of CTs performed in children with head injuries. In 2006, a clinical decision rule was derived in England for identification of children who should have CT done after their head injury—Children’s Head Injury Algorithm for the Prediction of Important Clinical Events, CHALICE [10]. In 2009, a study from the Pediatric Emergency Care Applied Research Network (PECARN) was published, with the aim to identify children at very low risk of CITBI in whom CT might be unnecessary [4, 11]. They derived and validated age-specific prediction rules for CITBI. Follow-up studies on the PECARN study show an absolute reduction of 6% in CT rate (from 21 to 15%) and good predictive performances of their clinical decision rule for minor head trauma in children [11, 12]. In 2010 a multicentre cohort study from Canada also addressed this matter and developed a decision rule for CT use in children with minor head injury – Canadian Assessment of Tomography for Childhood Head injury (CATCH) [13]. A revision of the CATCH guidelines, CATCH2, was published in 2018 [14].

These three decision rules—CHALICE, PECARN and CATCH, have been validated and shown to have high sensitivity [14, 15].

**Fig. 1:**
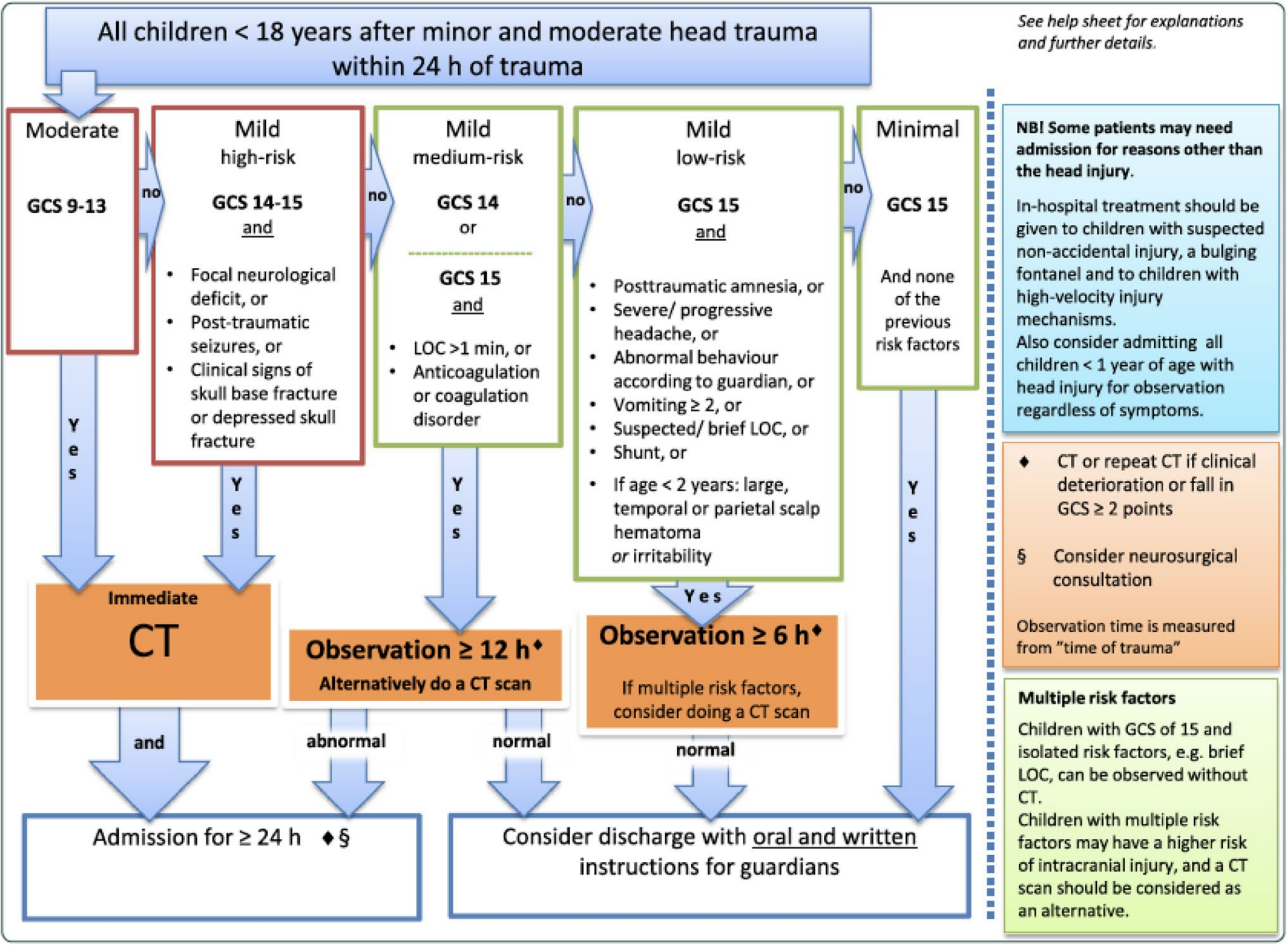
Flowchart for the management of children with minimal, mild, and moderate head injuries (GCS 9–15) according to the Scandinavian guidelines for initial management of minor and moderate head injuries in children (Astrand et al. [16])

Recently, the Scandinavian Neurotrauma Committee (SNC) published the Scandinavian guidelines for initial management of minor and moderate head trauma in children (SNC-G) [16]. The purpose of these guidelines is to assist physicians to determine which children need head CT and/or in-hospital observation, and who can be directly discharged from the emergency department. The SNC-G classifies head injuries in children as moderate, mild or minimal. The mild head injury is further subdivided into high-risk, medium-risk or low-risk injury. Depending on classification, different management is proposed (Fig. 1).

Our aim was to validate the SNC-G by assessing the risk of a child being discharged with a CITBI. A secondary aim was to assess the risk of a child being discharged with a TBI on CT.

## Methods

We did a retrospective cohort study of children with mild and moderate TBI to validate the SNC-G.

The cohort was obtained from the dataset of a prospective, observational multicentre study by Kuppermann et al.,: “Identification of children at very low risk of clinically-important brain injuries after head trauma: a prospective cohort study” run by PECARN [4, 17]. The aim of that study was to identify children with very low risk of CITBI, for whom CT might be unnecessary. Between June 2004 and September 2006, they enrolled 43 904 patients younger than 18 years presenting within 24 h of blunt head trauma in 25 North American emergency departments, mainly in paediatric hospitals. They recorded information about patient history, injury mechanism and symptoms and the data were compiled in a dataset. Children with trivial injury mechanisms, penetrating trauma, known brain tumours, ventricular shunts, bleeding disorders or pre-existing neurological disorders were not enrolled in the parent study.

Kuppermann et al. [4] defined CITBI as at least one of the following: hospitalisation for more than 2 nights associated with traumatic finding on CT, intubation for more than 24 h for traumatic brain injury, neurosurgical intervention, or death from traumatic brain injury. They defined traumatic brain injury on CT as any traumatic pathology found on CT.

For our cohort all children with GCS score 3–8 and/or with missing primary outcome, i.e. CITBI, were excluded.

The SNC-G contains several variables to assess in the management of head trauma in children. The dataset has corresponding variables for almost every one of these, with certain assumptions made a priori (Table 1).

**Table 1:** Variables in the SNC-G and the corresponding information collected from the PECARN

Variables in SNC-G	Information in PECARN dataset
GCS score	GCS score
Focal neurological deficit	Neurological deficit (other than mental status)
Post-traumatic seizures	Post-traumatic seizure
Clinical signs of skull base fracture or depressed skull fracture	Palpable skull fracture that feels depressed
	Signs of basilar skull fracture
LOC > 1 min	LOC > 1 min
Post-traumatic amnesia	Amnesia for the event
Severe/Progressive headache	Severe headache. Information about progressive headache is missing
Abnormal behaviour according to guardian	Does the parent think the child is acting normally/like themself?
Vomiting ≥ 2	≥ 2 vomiting episodes
Suspected/brief LOC	LOC < 1 min
	Suspected LOC
If age < 2 years: large, temporal or parietal scalp hematoma *or* irritability	Age < 2 years
	Large (diameter > 3 cm) of largest hematoma or swelling
	Hematoma(s) or swelling(s) involves parietal/temporal location
	Other signs of altered mental status: agitated
Anticoagulation or coagulation disorder	Not available
Shunt	Not available

*GCS* Glasgow Coma Scale, *LOC* loss of consciousness

This made it possible to categorise the children according to the SNC-G. Two variables in the SNC-G, the presence of ventricular shunts and bleeding disorders, are not to be found in the dataset since patients with these risk factors were excluded in their study and dataset [4]. Children with these conditions are therefore not included in the validation. All children in the present study were categorised into the SNC-G head injury severity groups; moderate, mild high-risk, mild medium-risk, mild low-risk and minimal (Fig. 1). The prevalence of CITBI and traumatic CT abnormalities were calculated and compared. Negative predictive values were calculated.

In the SNC-G, the management includes observation for 6 and 12 h. No information about the duration of observation were included in the dataset, and therefore no such data were analysed.

### Statistical analysis

Continuous variables were reported using means ± standard deviation. Categorical variables were reported using median and range. Difference between groups were analysed as applicable with Student’s *t *test and Chi-square test. Logistic regression was used to assess the association between the SNC-G variables and CITBI. In the logistic regression analysis all children with any missing variable were excluded. For statistical analysis, SPSS Statistics version 20 (IBM Corp, Armonk, NY, USA) and MedCalc (MedCalc Software, Ostend, Belgium) were used. A level of p < 0.05 was regarded as statistically significant.

### Ethics

The Regional Ethics Committee in Uppsala decided that no ethics approval was needed for this study (Dnr 2017/362). All data in the dataset are de-identified.

## Results

**Fig. 2:**
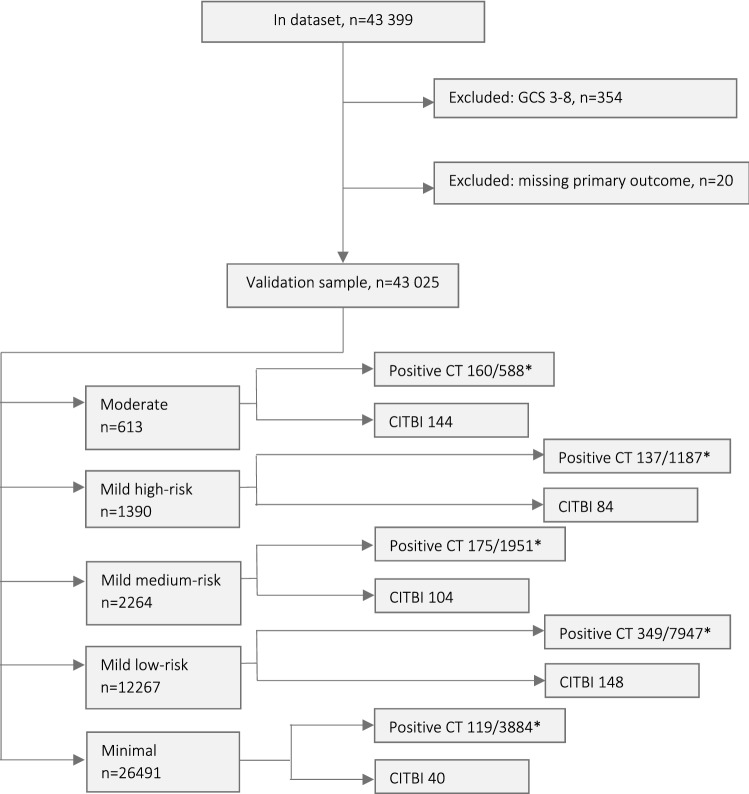
Study flowchart. *CITBI* clinically-important traumatic brain injury. *Positive CT scans/Total number of CT scans performed, in each group. Positive CT is defined as any traumatic finding on CT

Of 43 399 children in the dataset, 374 were excluded (Fig. 2). Among the 43 025 children eligible for analysis, 62.3% were males and the mean age were 6.6 years. A fourth of the children were younger than 2 years. Table 2 illustrates the characteristics of the study population and the distribution of the different guideline variables. Figure 2 also illustrates the categorisation of the children into the separate groups outlined by the SNC-G.

**Table 2:** Characteristics of study population and prevalence of SNC-G variables

		All	Girls	Boys	p*
Glasgow Coma Scale score					
Mean ± SD		14.9 ± 0.4	14.9 ± 0.4	14.9 ± 0.4	0.36^†^
Median (Range)		15 (9–15)	15 (9–15)	15 (9–15)	0.12^‡^
Age					
Mean ± SD		6.6 ± 5.5	6.0 ± 5.54	6.9 ± 5.5	** < 0.01**^**†**^
Median (range)		5 (0–17)	4 (0–17)	6 (0–17)	** < 0.01**^**‡**^
Focal neurological deficit		590/42,559 (1.4%)	235/16,030 (1.5%)	355/26,526 (1.3%)	0.28^‡^
Post-traumatic seizure		542/42,226 (1.3%)	192/15,939 (1.2%)	350/26,284 (1.3%)	0.26^‡^
Clinical signs of basilar skull fracture		329/42,596 (0.8%)	122/16,041 (0.8%)	207/26,552 (0.8%)	0.83^‡^
Depressed skull fracture		86/42,987 (0.2%)	34/16,197 (0.2%)	52/26,787 (0.2%)	0.80^‡^
Loss of consciousness					
Suspected		1979/41,191 (4.8%)	697/15,541 (4.5%)	1282/25,647 (5.0%)	**0.02**^**‡**^
> 1 min duration		1360/40,599 (3.3%)	428/15,398 (2.8%)	931/25,198 (3.7%)	** < 0.01**^**‡**^
Post-traumatic amnesia		4533/40,814 (11.1%)	1393/15,423 (9.0%)	3140/25,391 (12.4%)	** < 0.01**^**‡**^
Severe headache		822/41,984 (2.0%)	329/15,802 (2.1%)	493/26,179 (1.9%)	0.15^‡^
Abnormal behaviour according to guardian		6606/39,858 (16.6%)	2349/15,036 (15.6%)	4257/24,820 (17.2%)	** < 0.01**^**‡**^
Vomiting ≥ 2 times		3296/42,750 (7.7%)	1373/16,106 (8.5%)	1922/26,641 (7.2%)	** < 0.01**^**‡**^
Large hematoma or swelling^a^		3178/42,302 (7.5%)	996/15,941 (6.2%)	2182/26,361 (8.3%)	** < 0.01**^**‡**^
Age < 2 years and…					
Parietal/Temporal location of hematomas or swellings		1078/10,850 (9.9%)	627/5971 (10.5%)	451/4879 (9.2%)	**0.03**^**‡**^
Agitated		324/10,850 (3.0%)	186/5971 (3.1%)	138/4879 (2.8%)	0.40^‡^

*The *p *value of the difference between boys and girls in each variable. Bold numbers are statistically significant *p* values (< 0.05)

^a^A large hematoma/swelling is defined as > 3 cm

^†^Student’s *t* test

^‡^Chi-square-test

CT scans were obtained in 15,557 (36.2%) of all children in the study, of whom 940 (6.0%) had at least one traumatic finding on the CT scan. Totally, 520 (1.2%) had a CITBI, of whom 92 (17.7%) underwent neurosurgery and 39 (7.5%) were intubated for more than 24 h for traumatic brain injury. A total of 408 children did not need neurosurgery or intubation for more than 24 h but were hospitalized for more than 2 nights. No child died from their injury. Five children without any traumatic finding on CT had a CITBI, all intubated for more than 24 h and 1 in need of neurosurgery.

Table 3 illustrates the number of children in each SNC-G head injury group and the distribution between boys and girls within the groups. Of all head injuries, 98.6% were classified as minimal or mild and 1.4% as moderate.

**Table 3:** Classification into SNC-G head injury severity groups

	All *n* = 43,025^a^	Girls *n* = 16,207	Boys *n* = 26,815	p*
SNC-G group^b^				
Minimal	26,491 (61.6%)	10,121 (62.4%)	16,369 (61.0%)	** < 0.01**
Mild low-risk	12,267 (28.5%)	4576 (28.2%)	7690 (28.7%)	0.32
Mild medium-risk	2264 (5.3%)	769 (4.7%)	1494 (5.6%)	** < 0.01**
Mild high-risk	1390 (3.2%)	530 (3.3%)	860 (3.2%)	0.72
Moderate	613 (1.4%)	211 (1.3%)	402 (1.5%)	0.09

*SNC-G* Scandinavian guidelines for initial management of minor and moderate head trauma in children

*The *p* value of the difference between boys and girls in each group. Bold numbers are statistically significant *p* values (< 0.05)

^a^3 children with unknown sex

^b^Head injury severity group according to the SNC-G

As shown in Table 4 the prevalence of CITBI was lowest in the minimal head injury group. The prevalence was statistically significantly higher in the moderate group compared to the mild high-risk group (*p* < 0.001), in the mild medium-risk group compared to the mild low-risk (*p* < 0.001), and in the Mild low-risk group compared to the minimal group (*p* < 0.001). The prevalence of CITBI was not statistically significantly higher in the mild high-risk group compared to the mild medium-risk (*p* = 0.054).

**Table 4:** The prevalence of CITBI, traumatic findings on CT (percentage of all children with CT scan performed), neurosurgery and intubation > 24 h, in each head injury severity group

	CITBI (%)	CT + (%)	Neurosurgery (%)	Intubation^b^ (%)
Minimal (*n* = 26,491)	0.2	3.1	0.0^a^	0
Mild low-risk (*n* = 12,267)	1.2	4.4	0.2	0.0
Mild medium-risk (*n* = 2264)	4.6	9.0	0.4	0.2
Mild high-risk (*n* = 1390)	6.0	11.5	1.3	0.1
Moderate (*n* = 613)	23.5	27.2	5.2	5.1
All (*n* = 43,025)	1.2	6.0	0.2	0.1

*CITBI* clinically important traumatic brain injury, *CT +*  any traumatic finding on CT

^a^11 had surgery (0.04%)

^b^Intubation > 24 h

Of children with minimal head injury according to the SNC-G, 40 (0.2%) had a CITBI, 11 (0.04%) underwent neurosurgery, no one died, no one was intubated for more than 24 h, and 29 (0.1%) children were hospitalized for more than 2 nights without neurosurgical intervention or intubation for more than 24 h. Traumatic finding on CT was detected in 119 children in the minimal group, which accounts for 3.1% of all children who underwent a CT examination and 0.5% of all children in total in the minimal head injury group.

The reliability of the SNC-G is calculated as shown in Table 5. The SNC-G (minimal group *vs* mild and moderate groups) have a negative predictive value of 26,451/26,491 (99.9%) and sensitivity of 480/520 (92.3%) for CITBI. For traumatic findings on CT the negative predictive value is 3765/3884 (96.9%) and the sensitivity is 821/940 (87.3%). For the need of neurosurgery the negative predictive value is 26,480/26,491 (99.9%) and the sensitivity is 81/92 (88.0%).

**Table 5:** The ability of the SNC-G to distinguish children with risk of CITBI, TBI on CT and need of neurosurgery

Minimal vs Mild low-/medium-/high-risk + Moderate			
CITBI			
Head injury group	CITBI	No CITBI	Total
Mild low/medium/high-risk + moderate	480	16,054	16,534
Minimal	40	26,451	26,491
Total	520	42,505	43,025
Guideline sensitivity (95% CI)	92.3% (89.7–94.5)		
Guideline specificity (95% CI)	62.2% (61.8–62.7)		
Positive predictive value (95% CI)	2.9% (2.8–2.9)		
Negative predictive value (95% CI)	**99.9% (99.8–99.9)**		

TBI on CT			
Head injury group	CT +	CT-	Total
Mild low/medium/high-risk + moderate	821	10,852	11,673
Minimal	119	3765	3884
Total	940	14,617	15,557
Guideline sensitivity (95% CI)	87.3% (85.0–89.4)		
Guideline specificity (95% CI)	25.8% (25.1–26.5)		
Positive predictive value (95% CI)	7.0% (6.9–7.2)		
Negative predictive value (95% CI)	**96.9% (96.4–97.4)**		

Neurosurgery			
Head injury group	Neuro-surgery	No neuro-surgery	Total
Mild low/medium/high-risk + moderate	81	16,453	16,534
Minimal	11	26,480	26,491
Total	92	42,933	43,025
Guideline sensitivity (95% CI)	88.0% (79.6–93.9)		
Guideline specificity (95% CI)	61.7% (61.2–62.1)		
Positive predictive value (95% CI)	0.5% (0.5–0.5)		
Negative predictive value (95% CI)	**99.9% (99.9–99.9)**		

Minimal + Mild low-risk *vs* Mild medium-/high-risk + Moderate			
CITBI			
Head injury group	CITBI	No CITBI	Total
Mild medium/high-risk + moderate	332	3935	4267
Minimal + Mild low-risk	188	38,570	38,758
Total	520	42,505	43,025
Guideline sensitivity (95% CI)	63.9% (59.6–68.0)		
Guideline specificity (95% CI)	90.7% (90.5–91.0)		
Positive predictive value (95% CI)	7.8% (7.3–8.3)		
Negative predictive value (95% CI)	**99.5% (99.5–99.6)**		

TBI on CT			
Head injury group	CT +	CT-	Total
Mild medium/high-risk + moderate	472	3254	3726
Minimal + Mild low-risk	468	11,363	11,831
Total	940	14,617	15,557
Guideline sensitivity (95% CI)	50.2% (47.0–53.5)		
Guideline specificity (95% CI)	77.7% (77.1–78.4)		
Positive predictive value (95% CI)	12.7% (11.9–13.5)		
Negative predictive value (95% CI)	**96.0% (95.8–96.3)**		

Neurosurgery			
Head injury group	Neuro-surgery	No neuro-surgery	Total
Mild medium/high-risk + moderate	60	4207	4267
Minimal + Mild low-risk	32	38,726	38,758
Total	92	42,933	43,025
Guideline sensitivity (95% CI)	65.2% (54.6–74.9)		
Guideline specificity (95% CI)	90.2% (89.9–90.5)		
Positive predictive value (95% CI)	1.4% (1.2–1.6)		
Negative predictive value (95% CI)	**99.9% (99.9–99.9)**		

Bold numbers highlight the negative predictive values

*SNC-G* Scandinavian guidelines for initial management of minor and moderate head trauma in children, *TBI* Traumatic brain injury, *CITBI* clinically important TBI, *CI* confidence interval, *CT ±*  positive/negative finding on CT, i.e. any traumatic finding on CT

If we would discharge the children in the Mild low-risk group without the recommended observation time, i.e. merge the Mild low-risk group with the Minimal group, the negative predictive value for CITBI would be 38,570/38,758 (99.5%). The negative predictive values for TBI on CT and neurosurgery would be 11,363/11,831 (96.0%) and 38,726/38,758 (99.9%), respectively. As shown in Table 5, the sensitivity would be significantly reduced.

In the analysis of association between variables (gender, age and SNC-G variables) and CITBI, 9686 children were excluded because of missing data in any of the included variables. The 33,339 children included were analysed with logistic regression (Table 6). All these variables in the mild high-risk group had a statistically significant positive association to CITBI. The most powerful predictor of CITBI was the presence of clinical signs of skull base fracture or depressed skull fracture. Two variables in the SNC-G had not a statistically significant association to CITBI; post-traumatic amnesia and severe headache.

**Table 6:** Association between variables (age, gender and SNC-G variables) and CITBI

	No. (%) of patients		OR (95% CI)	p*
	CITBI	No CITBI		
	*n* = 180	*n* = 33,159		
Gender, male (v. female)	109 (60.6)	20,544 (62.0)	0.9 (0.6–1.2)	0.45
Age, year				
0	44 (24.4)	4553 (13.7)	1.0 (reference)	
1–5	52 (28.9)	13,762 (41.5)	0.3 (0.2–0.6)	** < 0.01**
6–9	29 (16.1)	5298 (16.0)	0.5 (0.2–1.2)	0.12
10–13	26 (14.4)	4727 (14.3)	0.5 (0.2–1.0)	0.06
14–17	29 (16.1)	4819 (14.5)	0.4 (0.2–0.9)	**0.02**
Glasgow Coma Scale score				
15	123 (68.3)	32,386 (97.7)	1.0 (reference)	
14	39 (21.7)	619 (1.9)	4.7 (3.0–7.3)	** < 0.01**
9–13	18 (10.0)	154 (0.5)	5.7 (3.0–10.8)	** < 0.01**
Post-traumatic amnesia (v. no post-traumatic amnesia)	32 (17.8)	2174 (6.6)	1.3 (0.8–2.1)	0.37
Severe headache (v. no, mild or moderate headache)	9 (5.0)	512 (1.5)	1.2 (0.6–2.6)	0.64
Abnormal behaviour according to guardian (v. normal behaviour)	108 (60.0)	4444 (13.4)	3.9 (2.7–5.6)	** < 0.01**
Vomiting ≥ 2 times (v. 0 or 1 time)	36 (20.0)	2428 (7.3)	1.9 (1.2–2.8)	** < 0.01**
LOC (v. no LOC)				
Suspected/brief LOC	24 (13.3)	2289 (6.9)	1.7 (1.1–2.9)	**0.03**
LOC > 1 min	33 (18.3)	837 (2.5)	4.0 (2.5–6.6)	** < 0.01**
Focal neurological deficit (v. no focal neurological deficit)	11 (6.1)	376 (1.1)	3.1 (1.5–6.3)	** < 0.01**
Post-traumatic seizures (v. no post-traumatic seizure)	11 (6.1)	321 (1.0)	2.5 (1.2–5.1)	**0.02**
Clinical signs of skull base fracture or depressed skull fracture (v. no signs of fracture)	31 (0.2)	211 (0.6)	26.5 (16.5–42.5)	** < 0.01**
Age < 2 years and: large, temporal or pariteal scalp hematoma or irritability (v. ≥ 2 years)	23 (12.8)	394 (1.2)	3.0 (1.4–6.2)	** < 0.01**
Age < 2 years without: large, temporal or parietal scalp hematoma or irritability (v. ≥ 2 years)	39 (21.7)	8812 (26.6)	0.7 (0.4–1.4)	0.36

*SNC-G *Scandinavian guidelines for initial management of minor and moderate head trauma in children, *CITBI* clinically-important traumatic brain injury, *CI* confidence interval, *OR* odds ratio, *LOC* Loss of consciousness.

*The *p *value of the OR for each variable. Bold numbers indicate a statistical significant *p* value (< 0.05)

## Discussion

We validated the SNC-G in a large, diverse population of children with minor and moderate head trauma. One of the most important parts of these guidelines is to recognise children who should be discharged from the emergency department without further examinations or observation i.e. children with minimal head injury. The results show a very high negative predictive value for CITBI (99.9%), i.e. ability to identify children without CITBI in this group. The SNC-G also has high sensitivity for the detection of CITBI (92.3%). These patients can thus be safely discharged with oral and written instructions for guardians, as the SNC-G recommends.

The SNC-G has a high negative predictive value for traumatic findings on CT (96.9%). This indicates that the risk of traumatic findings on CT for a child with a minimal head injury is low and suggests that CT scans in minimal head injured children could be omitted. Among all children enrolled in the study, 25.0% of the CTs were performed on children in this group. To some extent, by the favouring of short-term observation, unnecessary CT scans could also be avoided in the mild low-risk head injury group, which accounted for 51.1% of the CTs. The present population was clinically evaluated to need a CT scan according to local routines in 25 emergency departments in North America. These were mainly paediatric hospitals, with lower rates of CT use than general hospitals [18]. The reduction of unnecessary CT scans is, therefore, possibly even greater since most children seeking emergency care are assessed at general hospitals [4]. In the present study, CT scans were obtained in 36.2% of all children—a rate near average compared with similar studies [11, 13, 18, 19]. The reduction of CT use in head-injured children by evidence-based guidelines has been predicted and/or shown in previous studies [4, 11, 13, 14]. During 2005, 323 961 children in Sweden underwent a brain or head/neck CT scan [7]. With respect to the induction rate of lethal malignancy for cranial CTs in the paediatric population, a possible reduction of CT scans in children is of great importance.

The rate of TBI on CT scans in children with minimal and mild head trauma has in other studies ranged from 1–5% [20]. For mild, moderate and severe head traumas the rate of TBI on CT scan is naturally higher, in one study 8% [6]. This correlates well with the rate of 6.0% in the present study, consisting of head injuries of minimal, mild and moderate severity.

According to the guidelines children in the Mild low-risk group should be observed for at least 6 h before they are discharged. In the present study, we could not evaluate the effect of this 6 h-observation time. What we could evaluate, however, was how safe it would be to send these children home based on the first evaluation, i.e. merge the Mild low-risk group with the Minimal. The negative predictive values for Minimal and Mild low-risk group vs. Mild medium- and high-risk and Moderate group were calculated. The negative predictive value for CITBI was 99.5%, for CT 96.0% and for neurosurgery 99.9%. These values are slightly lower than for the minimal group only, but for CITBI and neurosurgery still very high, more than 99%. To refrain from performing CT-scan in this risk group as the SNC-G suggests can, therefore, be considered safe. The reduction of sensitivity when merging the Mild low-risk and Minimal group should, however, be noted and justifies the advice of observation time. Following the SNC-G recommendations and observing children with a Mild low-risk injury would have spared 76.1% of all CTs performed on this population.

There is no consensus regarding the definition of CITBI, but the rate of CITBI is often significantly lower than the rate of TBI on CT scans [20]. This is because minor CT findings without clinical importance might be excluded in the definition [4, 10, 21]. On the other hand, some brain injuries are not detected on CT but identifiable by other modalities, such as MRI or SPECT brain perfusion imaging [22, 23]. The possible need of hospital care without a positive CT scan emphasises the importance of a patient-oriented outcome measure, such as the one used by the PECARN study.

The study population in the present study is large, which allows sufficient statistical power. The characteristics of the study sample are comparable to that of similar studies [11, 24, 25].

The purpose of the SNC-G is to assist physicians in the initial management of children with head trauma as guidance to detect intracranial complications. The SNC-G are developed with the small risk of CITBI after minor head trauma balanced against the potentially harmful long-term effect of ionising radiation. The CITBI prevalence in the different head injury groups were statistically significant between the moderate head injury group compared to the mild high-risk group (*p* < 0.01), in the mild medium-risk group compared to the mild low-risk (*p* < 0.01), and in the mild low-risk group compared to the minimal group (*p* < 0.001). It was not statistically significant between the mild high-risk and mild medium-risk head injury groups. In total, this suggests that the SNC-G can predict the severity of head injury in children with GCS between 9 and 15.

The very high negative predictive value for the need of neurosurgery (99.9%) confirms a very low risk of the need for neurosurgical intervention in the minimal head injury group. Of all children with CITBI, 27.5% needed neurosurgery in the minimal head injury group and 39.4% in the other groups. The corresponding rate for intubation > 24 h were 0% in the minimal group and 8.1% in the other groups. This indicates that children in the minimal head injury group did not need intervention at the same rates as children in the more severe head injury groups. These results suggest that these CITBI, on group level, are less complicated or severe than the CITBI in the other head injury groups.

The association between the SNC-G variables and CITBI were statistically significant for all the variables except two; post-traumatic amnesia and severe headache. Severe headache is, however, merged with progressive headache in the SNC-G, the latter unavailable in the dataset of use. This result is, therefore, difficult to assess. Progressive headache has in previous studies been shown to be a powerful predictor of brain injury [13]. On the other hand, progressive headache is to the mind of the authors a difficult symptom to evaluate, especially in the paediatric population. The level of association between the SNC-G variables and CITBI are not in line with the SNC-G head injury severity-classification. For example, loss of consciousness > 1 min (categorized as a Mild medium-risk variable) has higher association than two of the three Mild high-risk variables. This indicates that a repositioning of the variables between the head injury severity-groups could have a possible positive effect on the SNC-G accuracy.

The SNC-G are complementary to the SNC adult head injury guidelines by using the same severity classification. In the adult guidelines, the use of the serum biomarker S100B is incorporated, as an attempt to reduce unnecessary CT scanning. S100B is highly expressed in the astroglial cells, with elevated levels in serum following traumatic brain injuries. The possible use of S100B as a predictor of abnormal head CT results in children has not yet been clarified [26, 27].

Recently, Undén et al. [28] performed a validation of the SNC-G on a cohort of children with TBI from ten Australian and New Zealand hospitals. They also compared the accuracy of SNC-G, CATCH, CHALICE and PECARN clinical decision rules, with the conclusion that the SNC-G showed high accuracy and compares well with the others.

### Limitations

This study has limitations. It is a North American study population, whilst the guidelines are aimed for a Scandinavian population. When calculating positive and negative predictive values, the values are highly dependent on the prevalence of the disease in the population. A lower prevalence will increase the negative predictive value, and a higher prevalence will reduce it. The generalizability could, therefore, be questioned. We believe, however, that the possible differences in population characteristics such as ethnicity has little, or no, impact on the rate of CITBI in the SNC-G’s head injury severity groups.

CT scans were obtained in children with, according to local guidelines, indication for this. In children with minimal head injury this accounted for 14.7%. Findings on CT could, therefore, be missed. One could also argue for this being a selected group of children with a possibly higher prevalence of a more severe injury, than in children considered as having no need for CT.

We could not evaluate the impact of observation time on the safety of these guidelines since observation time data were not registered in the used dataset. This defines our aspect of validation to include the management of children in the minimal head injury group only since the Mild head injury groups include observation.

The PECARN study by Kupperman et al. is one of 52 papers included by Astrand et al., in the making of the SNC-G. Astrand et al., did not use the PECARN dataset in their analysis, but they did consider Kupperman et al.’s conclusions, which are based on the dataset. The use of the PECARN dataset in the validation of the SNC-G is therefore not optimal.

## Conclusion

Based on negative predictive value and sensitivity, patients with blunt head trauma classified as a minimal head injury by the SNC-G can be safely, as is suggested in the guidelines, discharged with oral and written instructions for guardians. The SNC-G have a very high negative predictive value for CITBI (99.9%), i.e. ability to identify children without CITBI, and high sensitivity for detection of CITBI (92.3%).

Use of the SNC-G will potentially reduce the use of CT. Among all children enrolled in the study, 25.0% of the CTs were performed on children with minimal head injury. The SNC-G also has a high negative predictive value for traumatic findings on CT (96.9%), suggesting that CT scans in minimal head injured children could be omitted. To some extent, by the favouring of short-term observation, unnecessary CT scans could also be avoided in the mild low-risk head injury group, which accounted for 51.1% of the CTs. Thus, following the SNC-G could have spared 76.1% of all CTs performed on this population.

## Data Availability

The dataset supporting the conclusions of this article is available at PECARN repository, https://pecarn.org/studyDatasets/StudyDetails?studyID=4.

## Abbreviations

CITBI: Clinically important traumatic brain injury
CT: Computed tomography
GCS: Glasgow Coma Scale
LOC: Loss of consciousness
PECARN: Pediatric emergency care applied research network
SNC: Scandinavian neurotrauma committee
SNC-G: Scandinavian Neurotrauma Committee Guidelines: Scandinavian guidelines for initial management of minor and moderate head trauma in children
TBI: Traumatic brain injury
